# Discovery of two new *Phaeoclavulina* species (Basidiomycota, Gomphaceae) from high-elevation habitats in the Qinghai–Xizang Plateau

**DOI:** 10.3897/mycokeys.128.174768

**Published:** 2026-02-09

**Authors:** Yong Shan, Xue-Lian Wu, Sheng-Bang Zhang, Yu-Qing Hou, Jin-Ping Guo, Wan-Lin Zhao, De-Ning Zhang, Zhao-Xiang Zhu, Shu-Yan Liu

**Affiliations:** 1 College of Plant Protection, Jilin Agricultural University, No. 2888 Xincheng Street, Changchun 130118, Jilin Province, China College of Plant Protection, Jilin Agricultural University Changchun China; 2 Engineering Research Center of Edible and Medicinal Fungi, Ministry of Education, Jilin Agricultural University, No. 2888 Xincheng Street, Changchun 130118, Jilin Province, China Engineering Research Center of Edible and Medicinal Fungi, Jilin Agricultural University Changchun China; 3 Qinghai Shanshui Natural Resources Survey Institute, Xining 810008, Qinghai Province, China Qinghai Shanshui Natural Resources Survey Institute Xining China; 4 Forestry and Grassland Station of Ping’an District, No. 194 Xinping Avenue, Ping’an Subdistrict, Ping’an District, Haidong 810600, Qinghai Province, China Forestry and Grassland Station of Ping’an District Haidong China

**Keywords:** Coralloid fungi, macrofungi, phylogenetic analyses, Qinghai–Xizang Plateau, taxonomy

## Abstract

The genus *Phaeoclavulina* is characterized by coralloid basidiomata with complex branching patterns and diverse pigmentation. Here, two new species, *Phaeoclavulina fruticosa***sp. nov**. and *P. qinghaiensis***sp. nov**., were discovered in high-elevation coniferous forests (ca. 2870 m) on the Qinghai–Xizang Plateau, China. *Phaeoclavulina fruticosa* is characterized by compact, densely branched, caespitose basidiomata that turn blue when injured and by dacryoid to ellipsoid basidiospores with patched to subconical to verrucose ornamentations. *Phaeoclavulina qinghaiensis* is characterized by yellow to orange-yellow basidiomata that do not change color when bruised, and by broadly ellipsoid to oblong, densely verrucose basidiospores. Phylogenetic analyses based on concatenated ITS + LSU rDNA sequences place the two new species as distinct, well-supported lineages within *Phaeoclavulina*. This study further expands the diversity of *Phaeoclavulina* on the Qinghai–Xizang Plateau.

## Introduction

The genus *Phaeoclavulina* Brinkmann was coined by Brinkmann (1897) to accommodate coralloid fungi characterized by fleshy, often fragile basidiomata with varying degrees of branching and ochraceous to light brown basidiospores, with *Phaeoclavulina macrospora* Brinkmann from Germany as the type species. Brinkmann (1897) also suggested that *Phaeoclavulina* exhibits the closest morphological affinities to *Clavaria testaceoflava* Bres., *Clavaria spinulosa* Pers., and *Clavaria abietina* Pers. Based on Brinkmann’s original circumscription, Van Overeem (1923) adopted the concept of *Phaeoclavulina*, transferred *Clavaria zippelii* Lév. to this genus as *Phaeoclavulina zippelii* (Lév.) Overeem. Subsequently, Corner (1970) accommodated species of *Phaeoclavulina* within the subgenus *Echinoramaria* Corner of *Ramaria* Fr. ex Bonord., a taxonomic treatment that was subsequently followed by Petersen (1981) in his comprehensive revision of *Ramaria*. The taxonomic concept of the genus underwent a major revision when Giachini (2004), based on an integrative analysis of morphological characters and molecular phylogenetic data, recognized *Phaeoclavulina* as a distinct genus within *Gomphus* sensu lato. This revised classification was further supported by subsequent molecular phylogenetic analyses (Giachini et al. 2010), which provided robust evidence for the recognition of *Phaeoclavulina* as an independent lineage within the Gomphaceae.

Taxonomically, the genus *Phaeoclavulina* is placed in the family Gomphaceae, (Gomphales, Agaricomycetes, Basidiomycota) (He et al. 2019). Species of *Phaeoclavulina* typically produce branched to merismatoid basidiomata, with coloration ranging from white, yellowish brown, and orange to green, olivaceous, bluish green, violet, brown, reddish cinnamon, brick red, and gray and are micromorphologically characterized by echinulate or verrucose basidiospores, basidia bearing two to four sterigmata, and a monomitic hyphal system with frequent clamp connections and gloeoplerous hyphae (Krishnapriya 2023). In 2025, several new species were described, including *Phaeoclavulina aurantilaeta* P.T. Deng & P. Zhang (Deng et al. 2025), *Phaeoclavulina aena* Pelissero, Kuhar & Nouhra, *Phaeoclavulina angularis* Pelissero, Kuhar & Nouhra, *Phaeoclavulina prasina* Pelissero, Kuhar & Nouhra and *Phaeoclavulina stelligera* Pelissero, Kuhar & Nouhra (Pelissero et al. 2025), as well as *Phaeoclavulina aurea* Y. Gao, X. Tong & C.L. Hou and *Phaeoclavulina fulva* Y. Gao, X. Tong & C.L. Hou (Tong et al. 2025). These newly described species provide further evidence supporting the current circumscription of the genus.

At present, 68 species of *Phaeoclavulina* have been recorded globally (Species Fungorum 2025, https://www.speciesfungorum.org/Names/Names.asp), with 25 species reported from China (Teng 1963; Zhang et al. 2005; Li 2008; Liu et al. 2022; Deng et al. 2024, 2025; Zheng et al. 2024; Tong et al. 2025). Members of the genus *Phaeoclavulina* primarily inhabit soil or decaying wood substrates and are predominantly distributed in tropical and subtropical regions, although occurrences in temperate zones have also been recorded (Giachini 2004; Deng et al. 2024).

The Qinghai–Xizang Plateau (QXP) is the highest plateau in the world and is characterized by unique geographical features and highly diverse climatic conditions, which have given rise to exceptionally rich and highly endemic biodiversity (Cao et al. 2025; Wang et al. 2025b). In recent years, the QXP has become a hotspot for the discovery of new macrofungal taxa, highlighting its remarkable but still underexplored fungal diversity. In the year 2025 alone, multiple novel fungal species discovered on the QXP have been reported. Liu et al. (2025) described *Entoloma griseopileum* M.Q. He & X. Liu from Qinghai Province; Han et al. (2025) described four new *Conocybe* species from Xizang; Lei et al. (2025) introduced two new species of *Pseudohydnum* from Xizang; Wang et al. (2025a) reported 15 new species belonging to six orders, 11 families, and 12 genera of Agaricomycetes (Basidiomycota) from the southern border of Xizang; and another study by Wang et al. (2025b) revealed 64 novel taxa of macrofungi from the QXP and its surrounding areas. These findings collectively underscore the exceptional fungal richness and endemism of the QXP, emphasizing the urgent need for continued taxonomic and ecological investigations in this unique high-altitude ecosystem.

Although taxonomic research on the genus *Phaeoclavulina* has increased in recent years, its species diversity on the QXP remains poorly studied, and many unknown taxa are likely to exist in this region. Two previously undocumented species of *Phaeoclavulina* were collected during the field investigations in Qinghai Province in 2024 and are described as two new species based on morphology and phylogenetic analysis.

## Materials and methods

### Specimen collection

In September 2024, three specimens of *Phaeoclavulina* were collected by the authors from QXP, China. Field photographs were taken in situ prior to collection, capturing the shape, branching structure, and color characteristics of the basidiomata under natural light. Close-up images were also obtained to document diagnostic features such as branching patterns, surface textures, and color changes upon bruising. All photographs were taken using a digital camera with standardized settings to ensure consistent color representation. Fresh specimens were dried at 45–50 °C on the same day of collection, sealed in plastic bags containing silica gel desiccant. Voucher specimens are deposited in the Herbarium of Mycology of Jilin Agricultural University (HMJAU), China.

### Morphological observation

Morphological identification was conducted based on both macroscopic and microscopic characteristics. Macroscopic features were primarily assessed using detailed field notes and photographs of fresh basidiomata taken at the time of collection. Color descriptions followed the standard color codes of Kornerup and Wanscher (1978). Sections of dried basidiomata were mounted in 5% KOH solution for the observation of internal tissue structures. Microscopic features, including basidiospores, basidia, and hyphae, were examined from dried material under a light microscope (Zeiss Axio Scope. A1, Germany).

To observe the surface ornamentation of basidiospores, dried spores were mounted on aluminum stubs with conductive carbon adhesive and coated with an approximately 10 nm layer of gold using a sputter coater (JEC-3000FC, JEOL Ltd., Japan), and then were observed with a field-emission scanning electron microscope (SEM; JSM-IT800, JEOL Ltd., Japan).

For each specimen, at least 30 mature basidiospores were randomly selected and measured. Spore dimensions were presented as (a)b–c(d), where b–c represents the 90% confidence interval (mean ± 1.64 × standard error), and a and d denote the extreme minimum and maximum values, respectively, and Qm represents the average Q value of all measured basidiospores ± the sample standard deviation (Liu et al. 2022).

### DNA extraction, PCR amplification, and sequencing

Genomic DNA was extracted from 20 mg of dried fungal tissue, which was ground into a fine powder in liquid nitrogen, using the Hi-DNAsecure Plant Kit (Tiangen Biotech, Beijing, China) according to the manufacturer’s instructions. The internal transcribed spacer (ITS) and the large subunit (LSU) regions of the nuclear rDNA were amplified using the primer pairs ITS4/ITS5 (White et al. 1990; Gardes and Bruns 1993) and LR5/LR0R (Vilgalys and Hester 1990). PCR amplification was performed in a final reaction volume of 25 µL, containing 12.5 µL of Premix Taq [TaKaRa Taq (1.25 U/25 µL), 2 × dNTP mixture (0.4 mM each), and 2 × Taq buffer with 3 mM Mg^2+^] (TaKaRa, Tokyo, Japan), 9.5 µL of double-distilled water (ddH_2_O), 1 µL of each primer, and 1 µL of genomic DNA. The PCR program consisted of an initial denaturation at 94 °C for 4 min, followed by 32 cycles of denaturation at 94 °C for 40 s, annealing at 55 °C (for ITS) or 52 °C (for LSU) for 40 s, and extension at 72 °C for 1 min. A final extension was performed at 72 °C for 8 min, followed by storage at 4 °C (Deng et al. 2024). PCR products were visualized by electrophoresis on a 1.2% agarose gel, and high-quality amplicons were purified and sequenced by Sangon Biotech Co., Ltd. (Shanghai, China). Forward and reverse sequences were assembled into consensus sequences using DNAMAN version 9.0.1.116 (Lynnon Corporation), and all assembled sequences were manually checked to ensure accuracy. BLASTn searches were conducted in the NCBI nucleotide database to confirm sequence identity and assess similarity to previously published *Phaeoclavulina* species.

### Alignment and phylogenetic analyses

The newly generated and validated sequences in this study were deposited in the GenBank database (Table 1). Based on previous studies (Deng et al. 2024, 2025; Zheng et al. 2024; Pelissero et al. 2025; Tong et al. 2025), available ITS and LSU sequences of 49 *Phaeoclavulina* species were retrieved from GenBank. *Ramaria admiratia* R.H. Petersen and *Ramaria calvodistalis* R.H. Petersen were selected as outgroup taxa based on their phylogenetic position (Zheng et al. 2024). The final ITS+LSU dataset, comprising 86 taxa (65 ITS and 71 LSU; Table 1), was used for multigene phylogenetic analyses. Multiple sequences were aligned using MUSCLE implemented in MEGA X (Kumar et al. 2018) and manually adjusted in BioEdit version 7.0.5.3 (Hall 1999).

**Table 1.** Specimen information and GenBank accession numbers of newly obtained sequences in this study.

Taxon	Voucher	GenBank accession numbers		Geographical origin	References
		ITS	LSU		
*Phaeoclavulina abietina* ^†^	OSC 134649	JX310378	JX287478	USA	Unpublished
*Phaeoclavulina abietina* ^†^	OSC 140661	JX310379	JX287479	USA	Unpublished
*Phaeoclavulina aena*	DP387	PQ474782	PQ453527	Argentina	Pelissero et al. (2025)
*Phaeoclavulina aena*	DP392	PQ474781	PQ453526	Argentina	Pelissero et al. (2025)
*Phaeoclavulina aeruginea*	MHHNU8909	ON262784	ON262781	China	Liu et al. (2022)
*Phaeoclavulina aeruginea*	MHHNU6887	ON262785	ON262782	China	Liu et al. (2022)
*Phaeoclavulina africana*	TENN39621	–	AY574653	USA	Giachini et al. (2010)
*Phaeoclavulina alboapiculata*	AMB 18590	MT055971	MT053248	Italy	Franchi and Marchetti (2022)
*Phaeoclavulina alboapiculata*	AMB 18585	MT055964	–	Italy	Franchi and Marchetti (2022)
*Phaeoclavulina alboapiculata*	AMB 18613	MT452509	–	Italy	Franchi and Marchetti (2022)
*Phaeoclavulina angularis*	DP031	PQ474790	PQ453547	Argentina	Pelissero et al. (2025)
*Phaeoclavulina angularis*	DP386	PQ474787	PQ453529	Argentina	Pelissero et al. (2025)
*Phaeoclavulina arcosuensis*	AMB 18532	MT055916	MT053207	Italy	Unpublished
*Phaeoclavulina aurantilaeta*	MHHNU 11750	PQ589930	PQ579885	China	Deng et al. (2025)
*Phaeoclavulina aurantilaeta*	MHHNU 12213	PQ589931	PQ579886	China	Deng et al. (2025)
*Phaeoclavulina aurea*	BJM 344955	PQ287856	PQ287860	China	Tong et al. (2025)
*Phaeoclavulina aurea*	BJTC L007	PQ287853	PQ287858	China	Tong et al. (2025)
*Phaeoclavulina bicolor*	MHHNU10702	PP809798	PP800475	China	Deng et al. (2024)
*Phaeoclavulina bicolor*	MHHNU10703	PP809799	PP800476	China	Deng et al. (2024)
*Phaeoclavulina carovinacea*	AMB 18533	NR_176719	–	Italy	Franchi and Marchetti (2022)
*Phaeoclavulina carovinacea*	AMB 18551	MT055933	–	Italy	Franchi and Marchetti (2022)
*Phaeoclavulina carovinacea*	AMB 18534	MT055918	–	Italy	Franchi and Marchetti (2022)
*Phaeoclavulina caroviridula*	AMB 18535	NR_177141	MT053208	Italy	Franchi and Marchetti (2022)
*Phaeoclavulina caroviridula*	AMB 18536	MT055920	MT053209	Italy	Unpublished
*Phaeoclavulina cinnamomea*	MHHNU10376	ON262786	ON262783	China	Liu et al. (2022)
*Phaeoclavulina clavarioides*	PRM:945441	LR723647	–	Czech	Kříž et al. (2019)
*Phaeoclavulina clavarioides*	PRM:945440	LR723646	LR723645	Czech	Kříž et al. (2019)
*Phaeoclavulina coniferarum*	AMB 18562	MT055942	–	Italy	Unpublished
*Phaeoclavulina coniferarum*	AMB 18531	NR_176718	NG_088119	Italy	Unpublished
*Phaeoclavulina corrugata*	SJ99002	–	AY586707	Sweden	Larsson et al. (2004)
*Phaeoclavulina curta*	AMB 18641	MW115423	MW092704	Italy	Franchi and Marchetti (2022)
*Phaeoclavulina curta* ^†^	AMB 18605	MT452501	–	Italy	Franchi and Marchetti (2022)
*Phaeoclavulina curta* ^†^	UBC F32034	KX236126	–	Canada	Unpublished
*Phaeoclavulina curta* ^†^	HAY-F-000746	PP294846	–	USA	Unpublished
*Phaeoclavulina cyanocephala*	TH9064	KT339249	KT339290	Guyana	Unpublished
*Phaeoclavulina decolor*	FH1	–	AY577843	USA	Unpublished
*Phaeoclavulina echinoflava* ^†^	HKAS 45984	PP809801	PP800478	China	Deng et al. (2024)
*Phaeoclavulina echinoflava* ^†^	HKAS 45992	PP809800	PP800477	China	Deng et al. (2024)
*Phaeoclavulina eumorpha*	TENN36218	–	AY574712	USA	Giachini et al. (2010)
*Phaeoclavulina flaccida* ^†^	AMB n.18209	MF288928	MF288936	Italy	Unpublished
*Phaeoclavulina flaccida* ^†^	AMB 18544	MT055926	MT053213	Italy	Unpublished
***Phaeoclavulina fruticosa***	**HMJAU60999**	**PV821750**	**PV822043**	**China**	**This study**
*Phaeoclavulina fulva*	BJTC C274	PQ287852	PQ287857	China	Tong et al. (2025)
*Phaeoclavulina fulva*	BJTC ZH0015	PQ287854	–	China	Tong et al. (2025)
*Phaeoclavulina fulva*	BJTC ZH1138	PQ287855	PQ287859	China	Tong et al. (2025)
*Phaeoclavulina gigantea*	FH109	–	AY574703	USA	Giachini et al. (2010)
*Phaeoclavulina grandis*	BR079158-06	–	AY574678	USA	Giachini et al. (2010)
*Phaeoclavulina guyanensis*	FH84	–	AY574706	USA	Giachini et al. (2010)
*Phaeoclavulina insignis*	FH104	–	AY574704	USA	Giachini et al. (2010)
*Phaeoclavulina jilinensis*	MHHNU9149	PP809802	PP800479	China	Deng et al. (2024)
*Phaeoclavulina jilinensis*	MHHNU9164	PP809803	PP800480	China	Deng et al. (2024)
*Phaeoclavulina jilinensis*	MHHNU10504	PP809804	PP800481	China	Deng et al. (2024)
*Phaeoclavulina liliputiana*	3281	–	MT214488	Mexico	González-Ávila et al. (2020)
*Phaeoclavulina liliputiana*	3533	–	MT214489	Mexico	González-Ávila et al. (2020)
*Phaeoclavulina liliputiana*	3266	–	MT214490	Mexico	González-Ávila et al. (2020)
*Phaeoclavulina liliputiana*	3563	–	MT214491	Mexico	González-Ávila et al. (2020)
*Phaeoclavulina longicaulis*	TENN33826	–	AY574700	USA	Giachini et al. (2010)
*Phaeoclavulina macrospora*	AMB 18614	MT452510	–	Italy	Franchi and Marchetti (2022)
*Phaeoclavulina minutispora*	AMB 18588	MT055969	MT053246	Italy	Franchi and Marchetti (2022)
*Phaeoclavulina minutispora*	AMB 18586	MT055965	MT053243	Italy	Franchi and Marchetti (2022)
*Phaeoclavulina murrillii*	AH:48382	MH322683	–	Spain	Martín et al. (2020)
*Phaeoclavulina mutabilis*	TENN39893	–	AY577838	USA	Unpublished
*Phaeoclavulina myceliosa*	AGK 035	JQ408230	–	USA	Unpublished
*Phaeoclavulina nigricans*	AMB 18589	MT055970	MT053247	Italy	Franchi and Marchetti (2022)
*Phaeoclavulina ochracea*	AMB 18542	MT055924	MT053211	Italy	Unpublished
*Phaeoclavulina pancaribbea*	TENN31836	–	AY574707	USA	Giachini et al. (2010)
*Phaeoclavulina prasina*	DP452	PQ474794	PQ453548	Argentina	Pelissero et al. (2025)
*Phaeoclavulina prasina*	DP454	PQ474792	PQ453543	Argentina	Pelissero et al. (2025)
*Phaeoclavulina prasina*	DP465	PQ474793	PQ453544	Argentina	Pelissero et al. (2025)
*Phaeoclavulina pseudozippelii*	BBH 43575	MG214661	MG214663	Thailand	Wannathes et al. (2018)
*Phaeoclavulina pseudozippelii*	BBH 43576	MG214660	MG214662	Thailand	Wannathes et al. (2018)
***Phaeoclavulina qinghaiensis***	**HMJAU60997**	**PV821748**	**PV822041**	**China**	**This study**
***Phaeoclavulina qinghaiensis***	**HMJAU60998**	**PV821749**	**PV822042**	**China**	**This study**
*Phaeoclavulina roellinii*	PRM:945445	LR723649	–	Czech	Kříž et al. (2019)
*Phaeoclavulina stelligera*	DP162	PQ474784	PQ453541	Argentina	Pelissero et al. (2025)
*Phaeoclavulina stelligera*	DP197	PQ474783	PQ453540	Argentina	Pelissero et al. (2025)
*Phaeoclavulina stelligera*	DP224	–	PQ453539	Argentina	Pelissero et al. (2025)
*Phaeoclavulina tropicalis*	NY551	–	AY577841	USA	Unpublished
*Phaeoclavulina viridis*	FH1853	–	AY574676	USA	Giachini et al. (2010)
*Phaeoclavulina viridis*	OSC 97708	–	AY574675	USA	Giachini et al. (2010)
*Phaeoclavulina yunnanensis*	HKAS 127150	OQ755411	PQ376602	China	Zheng et al. (2024)
*Phaeoclavulina yunnanensis*	HKAS 128154	OQ755412	PQ376603	China	Zheng et al. (2024)
*Phaeoclavulina zealandica*	PDD:43383	–	AY577849	New Zealand	Unpublished
*Phaeoclavulina zippelii*	FH2	–	AY577844	USA	Unpublished
*Ramaria admiratia*	TENN69114	NR_137862	NG_059504	USA	Hughes et al. (2014)
*Ramaria calvodistalis*	TENN69095	KJ416132	KJ416135	USA	Hughes et al. (2014)

“–” indicates sequence data not available or not submitted to GenBank for the respective locus. The sequences newly generated in this study are highlighted in bold.† Indicates species reported from the QXP in China.

Phylogenetic relationships were inferred using three complementary approaches: maximum parsimony (MP), maximum likelihood (ML), and Bayesian inference (BI). MP analyses were performed in PAUP* v4.0 (Swofford 2002) using a heuristic search with 100 random sequence additions. Tree statistics, including consistency index (CI), retention index (RI), and rescaled consistency index (RC), were calculated. ML analyses were performed using RAxML, implemented in raxmlGUI 2.0 (Edler et al. 2020), using the GTRGAMMA model with 1000 bootstrap replicates (Felsenstein 1985). BI analyses were conducted in MrBayes v3.2.7 (Ronquist et al. 2012), with the best-fitting nucleotide substitution model selected using MrModeltest v2.0 (Nylander 2004). Two parallel Markov chain Monte Carlo (MCMC) runs were executed for 1 million generations, sampling every 1000 generations and discarding the first 25% as burn-in. Convergence was assumed when the average standard deviation of split frequencies fell below 0.01. The topologies obtained from MP, ML, and BI analyses were congruent. The MP tree was selected for presentation, with branch lengths representing the number of inferred nucleotide substitutions. Bootstrap support values derived from MP, ML, and BI analyses were simultaneously mapped onto the corresponding nodes of the MP topology.

## Results

### Phylogenetic analyses

The concatenated ITS+LSU alignment matrix consisted of 1854 characters, of which 1102 positions (59.4%) were parsimony-informative, and 110 positions (5.9%) were parsimony-uninformative. *Ramaria admiratia* and *R. calvodistalis* were designated as outgroup taxa to root the phylogenetic tree.

**Figure 1.**
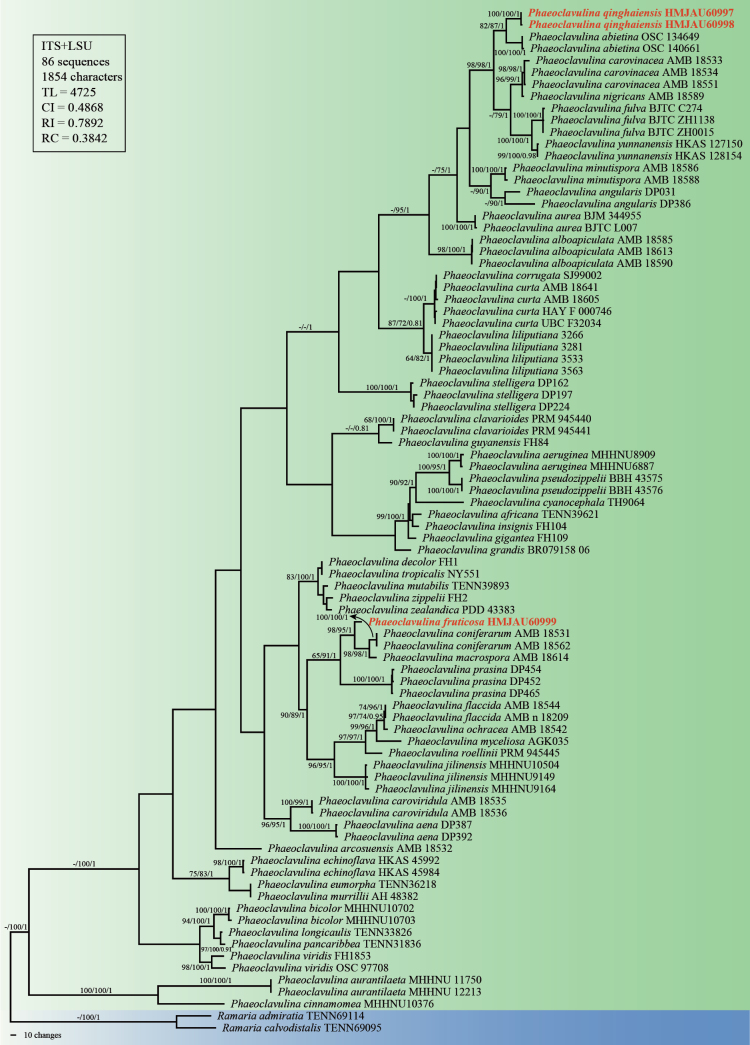
Phylogenetic tree of *Phaeoclavulina* species based on concatenated ITS and LSU sequences. Branches show bootstrap support values > 60% from maximum parsimony (MP) and maximum likelihood (ML) analyses, followed by posterior probabilities ≥ 0.70 from Bayesian inference (BI) analyses; “-” indicates support values below the respective threshold. Newly described species in this study are shown in red bold.

Phylogenetic analyses using MP, ML, and BI methods consistently formed a monophyletic clade comprising all *Phaeoclavulina* taxa (Fig. 1). *Phaeoclavulina fruticosa* (HMJAU60999) was formed as sister to a subclade comprising *Phaeoclavulina coniferarum* Franchi & M. Marchetti and *P. macrospora*, with nodal support values (MP/ML/BI = 98%/ 95%/ 1.00). This phylogenetic placement indicates that *P. fruticosa* is not nested within any known species complexes and supports its genetic distinctiveness.

The two newly collected specimens of *Phaeoclavulina qinghaiensis* (HMJAU60997 and HMJAU60998) formed a distinct and well supported lineage (MP/ML/BI = 100%/100%/1.00). This lineage was formed as sister to *Phaeoclavulina abietina* (Pers.) Giachini, and the clade comprising *P. qinghaiensis* and *P. abietina* was in turn resolved as sister to a clade including *Phaeoclavulina carovinacea* Franchi & M. Marchetti, *Phaeoclavulina nigricans* E. Campo, Franchi & M. Marchetti, *P. fulva*, *Phaeoclavulina yunnanensis* W.H. Lu, D.G. Zheng, Karun. & Tibpromma. The relatively long branch length separating this lineage from its closest relatives indicates its phylogenetic distinctiveness.

### Taxonomy

#### Phaeoclavulina fruticosa

Taxon classificationFungiGomphalesGomphaceae

Y. Shan & S.Y. Liu sp. nov.

E16641DD-CAE2-5CE8-8276-1D9AB55C1E3B

860375

Fig. 2

##### Etymology.

From the Latin “*fruticosus*” refers to the densely branched, shrub-like appearance of the basidiomata.

##### Diagnosis.

Basidiomata compact, densely branched, caespitose, turning blue when injured. Basidiospores dacryoid to ellipsoid with patched to subconical to verrucose ornamentations.

##### Type.

China • Qinghai Province, Xining City, Datong County, 37°04'16"N, 101°48'01"E, alt. 2870 m, 11 September 2024, Yong Shan, Shu-Yan Liu, Xue-Lian Wu, Sheng-Bang Zhang, Wan-Lin Zhao and De-Ning Zhang, HMJAU60999, holotype. GenBank: nuc rDNA ITS1-5.8S-ITS2 = PV821750; nuc rDNA 28S = PV822043.

##### Basidiomata.

Medium-sized, (20–)30–100(–105) × (6–)10–20(–24) mm, erect, compact, overall shrub-like to distinctly coralloid, typically caespitose, arising from soil on moss-covered coniferous forest floor. Branches with up to 4–5 levels of division, predominantly dichotomous; cylindrical to slightly compressed, ascending, mostly parallel or slightly divergent, closely spaced, and gradually tapering toward the apices; apices 1–2 mm long, usually blunt and commonly bifurcate. Stipe short to moderately developed, 10–50 mm high, distinctly widening toward the branching point. Texture fleshy, slightly viscid; tissues turning blue when injured. Fresh basidiomata grayish-yellow to olive-gray overall, with paler branch apices, yellow to yellowish-brown; with age, the overall coloration becomes progressively darker, turning olive-brown to brown. Surface dry to slightly moist, smooth to minutely pruinose. Context firm and fleshy; odor indistinct. Upon drying, basidiomata moderately shrink yet retain overall morphology and branching pattern; color darkens to olive-brown, with apices often remaining pale yellowish-brown; surface becomes hardened and brittle.

**Figure 2.**
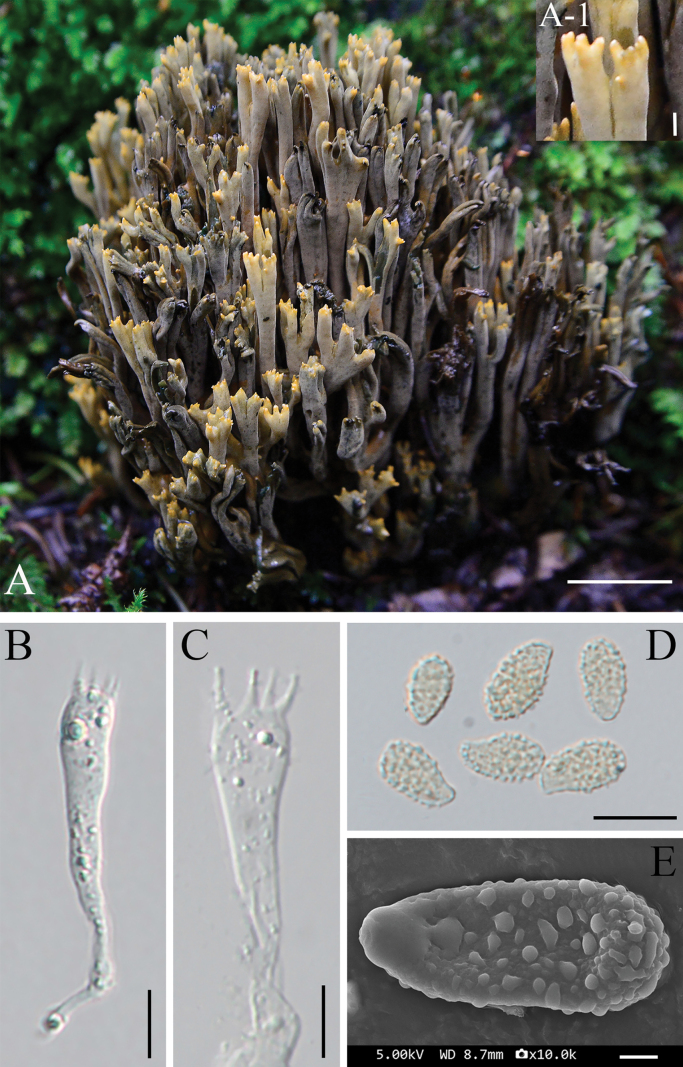
Basidiomata and microscopic features of *Phaeoclavulina fruticosa* sp. nov. **A**. Basidiomata; **A-1**. Enlarged view of the branch apex; **B, C**. Basidium; **D**. Basidiospores; **E**. Scanning micrograph of basidiospore. Scale bars: 2 cm (**A**); 2 mm (**A-1**); 10 μm (**B–D**); 1 μm (**E**).

##### Micromorphology.

Basidia 44.3–55.6 × 6.2–10.3 μm, slightly granular at maturity, hyaline to faintly yellowish, bearing four sterigmata. Hyphal system monomitic; hyphae hyaline, septate, with clamp connections, distinctly branched, (1.5–)2–6 μm wide, densely arranged, forming fasciculate or interwoven patterns.

Basidiospores (6.3–)7.4–8.0(–9.5) × (3.3–)4.1–4.5(–5.5) μm [Q = 1.45–2.16, Qm = 1.82 ± 0.2], dacryoid to ellipsoid, often slightly inequilateral, more or less rounded at one end and prolonged and sharp-pointed at the other; mature spores occasionally guttulate; surface roughened. Under SEM, basidiospores are distinctly ornamented, with surfaces sparsely covered by irregularly patched to subconical to verrucose ornamentations. The ornamentation elements are moderately sized, mostly discrete, and sparsely distributed across the spore surface, measuring approximately 0.15–0.35 µm in diameter. The ornamentations do not coalesce into ridges or form reticulate patterns.

##### Habit and distribution.

Gregarious on the soil around *Picea crassifolia* Kom. Known only from the type locality in Qinghai Province, China.

##### Notes.

*Phaeoclavulina fruticosa* forms a well-supported sister lineage to the *P. coniferarum* and *P. macrospora* clade. Despite this close phylogenetic relationship, *P. fruticosa* is readily distinguished from both species by a combination of macromorphological and micromorphological characters.

*Phaeoclavulina fruticosa* differs from *P. coniferarum* in having larger basidiomata (30–100 × 10–20 mm vs. 25–50 × 15–30 mm). With respect to fresh coloration and bruising reactions, basidiomata of *P. fruticosa* are grayish-yellow to olive-gray and turn blue when injured, while those of *P. coniferarum* are yellow to yellow-ochraceous and rapidly become green to cyan-green upon handling or exposure. Micromorphologically, *P. fruticosa* has larger basidia (44.3–55.6 × 6.2–10.3 μm) than *P. coniferarum* (35–45 × 5.5–7.5 μm). Furthermore, basidiospores of *P. fruticosa* are sparsely covered with irregularly patched to subconical to verrucose ornamentations, whereas *P. coniferarum* is characterized by densely spinose spores with spines reaching up to 0.5 μm in length.

*Phaeoclavulina fruticosa* differs from *P. macrospora* by its small basidiospores (7.4–8.0 × 4.1–4.5 μm vs. 14–20 × 4.5–8 μm). The basidiospore ornamentation also shows differences between the two species, spores of *P. macrospora* are prominently spinose, with spines reaching up to 2.5 μm in height, whereas spores of *P. fruticosa* are distinctly ornamented, with surfaces sparsely covered by irregularly patched to subconical to verrucose ornamentations (ca. 0.15–0.35 μm in diameter). The discoloration reactions upon injury are also different, with *P. macrospora* turning brown soon after bruising, whereas *P. fruticosa* turns blue.

#### Phaeoclavulina qinghaiensis

Taxon classificationFungiGomphalesGomphaceae

Y. Shan & S.Y. Liu sp. nov.

992554EE-06D4-525C-B3A3-B7E2EE6719D4

860374

Fig. 3

##### Etymology.

*Qinghaiensis* (Latin) refers to the type location, Qinghai Province, China.

##### Diagnosis.

Basidiomata yellow to orange-yellow, not changing color when bruised. Basidiospores are broadly ellipsoid to oblong and exhibit a dense covering of spherical to irregular warts.

##### Type.

China • Qinghai Province, Xining City, Datong County, 37°04'21"N, 101°49'04"E, alt. 2865 m, 11 September 2024, Yong Shan, Shu-Yan Liu, Xue-Lian Wu, Sheng-Bang Zhang, Wan-Lin Zhao and De-Ning Zhang, HMJAU60997, holotype. GenBank: nuc rDNA ITS1-5.8S-ITS2 = PV821748; nuc rDNA 28S = PV822041.

##### Basidiomata.

Arising from soil or moss-covered coniferous forest floor, typically gregarious or caespitose, erect, and distinctly coralloid in form, measuring (26–)30–60(–63) × (6–)10–20(–21) mm. Main branches thick, originating from a short stipe-like base and successively divided dichotomously or polychotomously, typically 4–5 times, with apices subacute, commonly forked or slightly recurved, measuring approximately 1.5–4 mm in length. Fresh basidiomata yellow to orange-yellow overall, paler at the base and slightly darker at the tips; color unchanging when bruised or injured. Surface dry, without gelatinous or tomentose covering; texture fleshy to firm. Stipe short and inconspicuous, central or slightly eccentric, often merging with the basal branches. In some specimens, basal stipe structures laterally connate, with distinct longitudinal grooves at the points of fusion, forming deeply embedded junctions. Basal parts frequently associated with rhizomorphic strands, sometimes bearing remnants of moss, pine needles, or cone scales. Upon drying, basidiomata dark brown to yellowish brown, with slight surface shrinkage but well-preserved branching structure; base pale yellowish white to light brown, with slightly fibrous texture.

**Figure 3.**
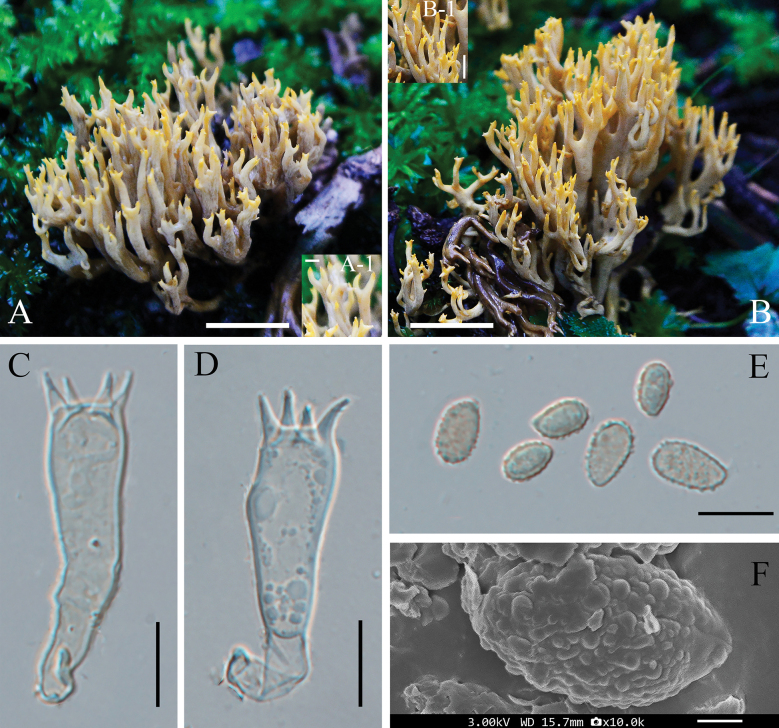
Basidiomata and microscopic features of *Phaeoclavulina qinghaiensis* sp. nov. **A, B**. Basidiomata; **A-1, B-1**. Enlarged view of the branch apex; **C, D**. Basidium; **E**. Basidiospores; **F**. Scanning micrograph of basidiospore. Scale bars: 2 cm (**A, B**); 2 mm (**A-1, B-1**); 10 μm (**C–E**); 1 μm (**F**).

##### Micromorphology.

Basidia 39.1–50.9 × 5.9–9.1 μm, slightly granular upon maturation, hyaline to faintly yellowish, bearing four sterigmata. Hyphal system monomitic; hyphae septate, with clamp connections; contextual hyphae (1.8–)2–5 μm wide, with variable wall thickness, branched, densely arranged.

Basidiospores (6.5–)8.4–8.9(–11.0) × (3.5–)4.8–5.3(–6.7) μm [Q = 1.19–2.31, Qm = 1.74 ± 0.27], broadly ellipsoid to oblong, slightly inequilateral, more or less rounded at one end and narrower and slightly prolonged at the other; surface distinctly roughened. Some spores contain one to several guttules. Under SEM, spores exhibit a dense covering of warty (verrucose) ornamentation. These ornamentations are spherical to irregular in shape, moderately sized (0.1–0.5 µm in diameter), evenly distributed, and do not coalesce into ridges or reticulate structures.

##### Notes.

*Phaeoclavulina qinghaiensis* formed as sister to *P. abietina* in the phylogenetic analyses, but the two species can be distinguished by several morphological characters. Basidiomata of *P. qinghaiensis* remain unchanged in color when bruised or injured, whereas the flesh of *P. abietina* becomes slightly ochraceous when cut. Basidiospores of *P. qinghaiensis* are broadly ellipsoid to oblong and often slightly inequilateral, while those of *P. abietina* are elongate and pip-shaped. In addition, differences are observed in basidiospore ornamentation: basidiospores of *Phaeoclavulina qinghaiensis* are verrucose, with spherical to irregular ornamentation that are evenly distributed and of moderate size (0.1–0.5 μm in diameter), whereas basidiospores of *P. abietina* bear closely echinulate ornamentation composed of short, acute, hyaline spines 0.5 μm long, or 0.5–1 μm long in other collections.

##### Additional materials examined.

China • Qinghai Province, Xining City, Datong County, 37°04'21"N, 101°49'04"E, alt. 2860 m, 11 September 2024, Yong Shan, Shu-Yan Liu, Xue-Lian Wu, Sheng-Bang Zhang, Wan-Lin Zhao and De-Ning Zhang, HMJAU60998, paratype. GenBank: nuc rDNA ITS1-5.8S-ITS2 = PV821749; nuc rDNA 28S = PV822042.

##### Habit and distribution.

Gregarious on the soil around *Picea crassifolia*. Known only from the type locality in Qinghai Province, China.

## Discussion

*Phaeoclavulina* was proposed by Brinkmann (1897) but remained controversial because many coralloid taxa with echinulate to verrucose basidiospores were traditionally placed in *Ramaria*, especially *Ramaria* subgenus *Echinoramaria* (Corner 1970). Subsequent integrative work has consistently recovered *Phaeoclavulina* as a distinct lineage, supporting its recognition as an independent genus (Giachini 2004; Giachini et al. 2010; Giachini and Castellano 2011). Morphologically, *Phaeoclavulina* species typically form coralloid (rarely subpileate) basidiomata with complex branching and variable pigmentation, and produce basidiospores that are usually echinulate to verrucose (Pelissero et al. 2025). The two new species in this study, *P. fruticosa* and *P. qinghaiensis*, conform to this concept and are placed in *Phaeoclavulina* by ITS–LSU phylogenetic analyses.

Species delimitation in *Phaeoclavulina* is supported by both molecular and morphological evidence. Two new species are formed as distinct, well-supported lineages in ITS and LSU analyses and can be separated from described species by stable morphological traits. A total of 68 species have been described in the genus *Phaeoclavulina*, bruise-induced discoloration and basidiospore ornamentation are essential characters for taxonomy. Of these, 16 species are reported to discolor after injury (Corner 1950; Giachini 2004; Franchi and Marchetti 2020; González-Ávila et al. 2020; Liu et al. 2022), and only eight species are characterized by predominantly verrucose spores (Giachini 2004; Zheng et al. 2024; Tong et al. 2025). *Phaeoclavulina fruticosa* is characterized by turning blue when bruised and irregularly patched to subconical to verrucose ornamentations of basidiospores. While *Phaeoclavulina qinghaiensis* is characterized by non-discoloring basidiomata and uniformly dense verrucae.

The QXP represents a habitat with distinctive environmental conditions and supports rich biodiversity, but *Phaeoclavulina* has been rarely studied there. Although *Phaeoclavulina* is widespread globally, records from the plateau are limited. To date, only 25 species have been reported from China. The discovery of *P. fruticosa* and *P. qinghaiensis* in high-elevation coniferous forests at approximately 2870 m in Qinghai Province further supports the view that the QXP harbors substantial but still poorly sampled diversity of *Phaeoclavulina*, and provides important insights into the presence of previously hidden species diversity within the genus. While the phylogenetic relationships of *Phaeoclavulina* at the genus level are well resolved, ecological differentiation and utilitarian traits among species remain incompletely known: *P. flaccida* is reported as toxic and inedible (Bau et al. 2024), whereas some ramarioid species currently placed in *Phaeoclavulina* are locally consumed in China (Li 2008; Dai et al. 2010), highlighting the need for more systematic studies of functional traits within the genus.

## Supplementary Material

XML Treatment for Phaeoclavulina fruticosa

XML Treatment for Phaeoclavulina qinghaiensis

## Additional information

### Use of AI

No use of AI was reported.

### Author ORCIDs

Yong Shan https://orcid.org/0009-0006-8040-5420

Zhao-Xiang Zhu https://orcid.org/0000-0002-8330-918X

Shu-Yan Liu https://orcid.org/0000-0001-9082-1053

### Data availability

All of the data that support the findings of this study are available in the main text.
